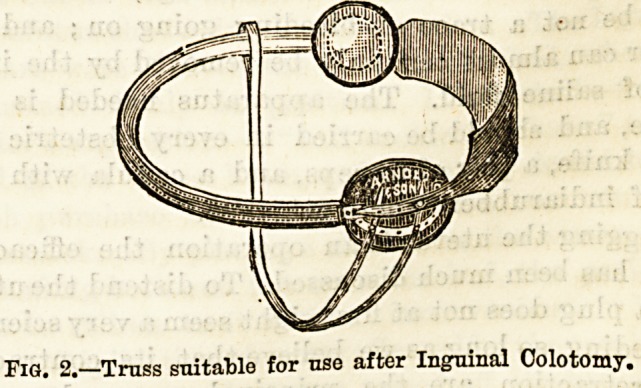# Inguinal Colotomy in Carcinoma of the Rectum

**Published:** 1894-03-31

**Authors:** W. McAdam Eccles

**Affiliations:** Assistant-Surgeon to the West London Hospital, Surgeon to the St. Marylebone General Dispensary, London, and Assistant-Demonstrator of Anatomy at St. Bartholomew's Hospital, Assistant Surgeon to the City of London Truss Society


### INGUINAL COLOTOMY IN CARCINOMA OF THE RECTUM.

By W. McAdam Eccles, M.B., B.S., F.R.C.S.Eng., Assistant-Surgeon to the West London Hospital, Surgeon to the St. Marylebone General Dispensary, London, and Assistant-Demonstrator of Anatomy at St. Bartholomew's Hospital, Assistant Surgeon to the City of London Truss Society.

Carcinoma of the rectum, or of the sigmoid flexure of the colon is unfortunately by no means a rare disease, and apparently on the increase with advancing civilization and its attendant evils of unnatural modes of life. Patients, moreover, are often attacked when outwardly in the best of health, and the growth may make considerable progress before any marked symptoms referable to the bowel arise. Thus it is that cases come under the notice of practitioners when it is hopeless to attempt any kind of radical treatment, and all that can be done is to alleviate suffering. Great relief can be procured in many cases by attention to diet, avoiding all articles of food which leave much detritus, prohibiting all spices and condiments, and making milk a prominent food. By enemata of oil and water carefully given a great deal of the irritation so frequent in the bowel may be lessened.

But a very large proportion of these rectal cases present sooner or later signs of intestinal obstruction, indistinct and chronic at first, but at any time becoming well marked and acute. It is an important question, therefore, and one which must be answered in every case, whether an opening into the bowel above the stricture should be made. Colotomy gives the patient the following advantages: (*a*) Safety from intestinal obstruction and all its accompanying misery; (*b*) a considerable relief from the pain and irritation produced by the passage of fæcal matter over the growth; (*c*) and probably some retardation in the rate of increase of the disease owing to the cessation of irritation, and possibly diminished blood supply. On the other hand against the performance of the operation of opening the colon are the following points: (*a*) The risk of the operation itself, which is but slight if done reasonably early, and with care; (*b*) the new discomfort of an artificial anus; (*c*) the certainty that no cure of the growth itself can result; (*d*) the probable continuance of some of the symptoms due to the disease; and (*e*), lastly, that a fair number will never have complete intestinal obstruction.

Ought patients, then, be urged to submit to colotomy when no definite symptoms of obstruction are present? No; but they should clearly understand that such symptoms may come on rapidly and quite unexpectedly, and that colotomy is imperative if all the signs of acute obstruction supervene; and, moreover, that the operation will then have to be undertaken under much less favourable circumstances. Complete obstruction in these cases is most commonly caused by a hard scybalous mass becoming impacted into the upper part of the strictured portion of the gut. In some cases it may be dislodged by a stream of liquid injected with some degree of force into the rectum, but if this treatment is not at once successful colotomy ought forthwith to be done. Again, if the patient is much worried by the incessant desire to defæcate, with little or no result, colotomy will usually give decided and immediate relief, thereby securing a happier existence for the necessarily short period of life that remains.

If colotomy is deemed advisable or absolutely necessary, either as a purely palliative measure or to afford relief from complete obstruction, where should the bowel be opened?

Inguinal, or iliac, colotomy has, to my mind, decided superiority over the lumbar operation, and chiefly because the bowel is almost always easily found; indeed, in many cases the sigmoid presents at the wound, and that the opening itself is made in a situation which is considerably more convenient for the patient subsequently than that in the loin. If the wounding of the peritoneum be urged as an objection to the inguinal method, a deliberate and planned incision through the abdominal wall into the peritoneal cavity is infinitely preferable to a possible or uncertain wound made in the lumbar region. Inguinal colotomy is certainly sometimes difficult, when there is much distension due to obstruction, and in these cases possibly lumbar colotomy is better. The mortality of inguinal colotomy of late years has not exceeded three per cent., whereas in lumbar colotomy it is still distinctly higher, though much less than formerly.

Inguinal colotomy, unless the symptoms are urgent, is performed in two stages. In the first, the abdominal walls are cut through in the left iliac region, across the junction of the outer third with the inner two-thirds of a line drawn from the umbilicus to the anterior superior spine of the ilium.

The incision is about three inches long, and one-half of it is on either side of the above point.

The sigmoid flexure, if it does not present, is usually easily found and drawn up into the wound by introducing the finger down into the left iliac fossa, and passing it upwards until it meets with the sigmoid mesocolon, which must lead to the sigmoid itself. The

Fig. 1.—A line of incision for Inguinal Colotomy.

gut is best stitched to the abdominal wall by many fine silk sutures, passing through only the serous and muscular coats, after the parietal peritoneum has been sutured with four stitches to the skin. The wound is then dressed aseptically, the intestine being covered with some protective, and left undisturbed for four or five days, if the patient's condition will allow of it. At the end of this time the gut is firmly fixed in its new position, and the second stage of the operation, that of opening the intestine, may be performed. This incision is quite painless, and no anæsthetic is needed. A good deal of hæmorrhage from the vessels in the wall of the bowel is to be expected, and may be arrested by ligatures.

It is well after the opening has been made to remove all scybalous masses and other detritus from the portion of the rectum above the stricture, chiefly by thorough washing out, so that there may be nothing left to cause irritation except the growth itself, the discharge from which is always likely to produce a certain amount of discomfort, of which the patient should be forwarned. If an efficient spur has been obtained, no more fæcal material will pass into the lower opening.

Subsequently patients develop a very fair control over their evacuations, and usually have a regular action of the bowel at their accustomed time in the day. A properly fitted special truss is all that is usually necessary to prevent leakage from the bowel, or prolapse of the mucous membrane. One designed by my colleague, Mr. H. J. Waring, M.S. (Lond.) is most suitable.

Fig. 2.—Truss suitable for use after Inguinal Colotomy.

The parts about the opening should be kept very clean by washing with soap and water, and any tendency to excoriation treated with a liberal application of zinc oxide ointment. It is an advantage to occasionally wash out the distal portion of the bowel both above and below the stricture.

Patients may live for many months in comparative comfort after the operation, who otherwise would have been doomed to a miserable existence, and the worst, perhaps, of all endings, that of complete intestinal obstruction.

## Figures and Tables

**Fig. 1. f1:**
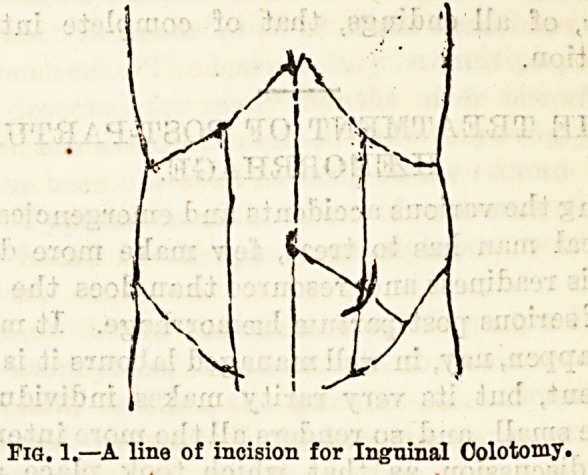


**Fig. 2. f2:**